# Ionic shape-morphing microrobotic end-effectors for environmentally adaptive targeting, releasing, and sampling

**DOI:** 10.1038/s41467-020-20697-w

**Published:** 2021-01-18

**Authors:** Zhiqiang Zheng, Huaping Wang, Lixin Dong, Qing Shi, Jianing Li, Tao Sun, Qiang Huang, Toshio Fukuda

**Affiliations:** 1 Intelligent Robotics Institute, School of Mechatronical Engineering, Beijing Institute of Technology, 100081 Beijing, China; 2 Beijing Advanced Innovation Center for Intelligent Robots and Systems, Beijing Institute of Technology, 100081 Beijing, China; 3 Department of Biomedical Engineering, City University of Hong Kong, 999077 Hong Kong, China; 4 Key Laboratory of Biomimetic Robots and Systems (Beijing Institute of Technology), Ministry of Education, 100081 Beijing, China

**Keywords:** Biomedical engineering, Mechanical engineering, Bioinspired materials

## Abstract

Shape-morphing uses a single actuation source for complex-task-oriented multiple patterns generation, showing a more promising way than reconfiguration, especially for microrobots, where multiple actuators are typically hardly available. Environmental stimuli can induce additional causes of shape transformation to compensate the insufficient space for actuators and sensors, which enriches the shape-morphing and thereby enhances the function and intelligence as well. Here, making use of the ionic sensitivity of alginate hydrogel microstructures, we present a shape-morphing strategy for microrobotic end-effectors made from them to adapt to different physiochemical environments. Pre-programmed hydrogel crosslinks were embedded in different patterns within the alginate microstructures in an electric field using different electrode configurations. These microstructures were designed for accomplishing tasks such as targeting, releasing and sampling under the control of a magnetic field and environmental ionic stimuli. In addition to structural flexibility and environmental ion sensitivity, these end-effectors are also characterized by their complete biodegradability and versatile actuation modes. The latter includes global locomotion of the whole end-effector by self-trapping magnetic microspheres as a hitch-hiker and the local opening and closing of the jaws using encapsulated nanoparticles based on local ionic density or pH values. The versatility was demonstrated experimentally in both in vitro environments and ex vivo in a gastrointestinal tract. Global locomotion was programmable and the local opening and closing was achieved by changing the ionic density or pH values. This ‘structural intelligence’ will enable strategies for shape-morphing and functionalization, which have attracted growing interest for applications in minimally invasive medicine, soft robotics, and smart materials.

## Abstract (web summary)

Making environmentally responsive materials often requires the use of multi-material devices. Here, the authors report on the use of electric field patterning of alginate hydrogels to make environmentally responsive actuators and demonstrate a range of applications in response to ionic triggers.

## Introduction

Living creatures, ranging from microorganisms to vertebrates and invertebrates, and even to some plants, use soft tissues to reversibly change their shapes for locomotion or object manipulation^1–4^. Although shape-morphing has become a commonplace for biomimicking microrobots, functional and intelligent embedding still remain a grand challenge to equip such small robots with multiple sensors and actuators that respond to external applied localized fields^5,6^. A variety of chemical^7,8^, physical^9–12^, and field^13–15^ stimuli have been successfully used to control morphology changes for locomotion or object manipulation using soft materials. However, because of the difficulty in localization of an external field, current designs are typically based on heterogeneous architectures^16–19^ and/or materials^20–24^ for implementing programmable pattern generation. It can be foreseen that environmental sensing and shape adaptivity are hurdles for achieving intelligent untethered microrobots designed for conducting tasks autonomously^25,26^. However, these methods can neither generate ion-induced shape morphing microrobotic end-effector using a single degradable biomaterial through integral forming technique, nor develop a multi-field-controlled robotic system which can conduct different locomotion patterns and accomplish automatic transformation in the respective physiochemical environments. Here, we present an ionic shape-morphing strategy for microrobots to adapt in physiochemical environments using alginate hydrogel microstructures, in which programmable hydrogel networks are spatially pre-embedded using an electric field, for accomplishing tasks, such as targeting, releasing, and sampling.

Despite the advances in biomimicry, natural morphing mechanisms still cannot be reproduced in artificial systems. For example, shape-morphing microstructures must be designed with heterogeneous features for non-uniform deformation, such as a bilayer structure that combines two types of hydrogel with different swelling rates^27,28^, or an inhomogeneous structure which achieves accurate spatial distribution of single material with different densities for the asymmetry in a whole architecture level^18,29^. These approaches compromise the properties of each hydrogel and complicate robotic miniaturization. Although controlled shape-morphing systems have been realized in soft^27^ and hybrid (soft and rigid)^30^ microarchitectures, deformation is typically wireless controlled using external sources, which limits the working environment in which the microrobot can operate. Thus, to create an intelligent shape-morphing microrobot, a heterogeneous architecture should be embedded into a single hydrogel so that the structure can undergo autonomous shape morphing under various physiochemical environments.

**Fig. 1:**
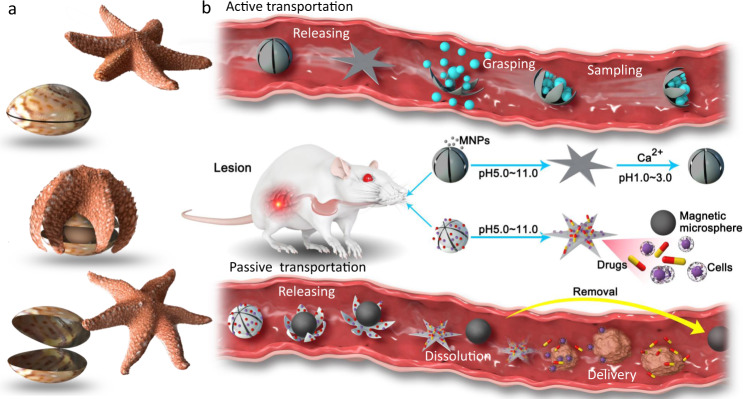
Inspiration for the ISME. **a** A starfish moves to, preys on, then moves away from a conch. **b** Schematic illustration of an ISME in the gastrointestinal tract. Under active transportation, the ISME releases a payload and conducts target sampling under enteric pH or ionic stimulus. Under passive transportation, the ISME releases a payload and dissolves under pH 5–11, allowing efficient delivery of drugs or cells to the intestines.

Here, we present entirely soft, shape-morphing alginate monolayers that can act as microscopic end-effectors to conduct grasp–release behavior in response to a pH change or an ionic shift. Microarchitectures are developed as ionic shape-morphing microrobotic end-effectors (ISMEs), which mimic the behaviors of starfish when they prey on conch, as illustrated in Fig. 1a. The starfish-shaped configuration is a suitable structure for grasp and release motions, which is able to tightly capture the objects and adapt various surface profiles. To achieve shape morphing for grasping and releasing stimulated by pH or ionic changes, the heterogeneous electrodeposition paves a way to embed an inhomogeneous hydrogel network into the alginate monolayer. Under ionically stimulated shrinkage or swelling of the alginate hydrogel, heterogeneous deformation of network leads to environmentally adaptive releasing or folding motion. In addition, we explore two strategies, active transportation, and passive transportation, to provide controllable locomotion and navigation to ISMEs under a magnetic field, as shown in Fig. 1b. To achieve active transportation, magnetic nanoparticles (MNPs) are encapsulated in the alginate microarchitecture. Passive transportation was achieved using self-trapping magnetic microspheres as hitch-hikers. When the ISME arrives at the target location, the magnetic microspheres separate from the ISME by self-release. One application of such an ISME is as a drug-delivery system, where the magnetic microspheres can be removed through excretion. The ISME could also be used as a sampling tool that can be propelled and navigated via the encapsulated MNPs. In addition, we demonstrate that ISMEs automatically conduct motions such as targeting, releasing, and sampling in response to the gastrointestinal environment. We believe that the developed ISMEs hold great potential for medical applications, such as targeted therapy, precise diagnostics, and tissue repair and regeneration.

## Results

### Fabrication and characterization of the ISMEs

**Fig. 2:**
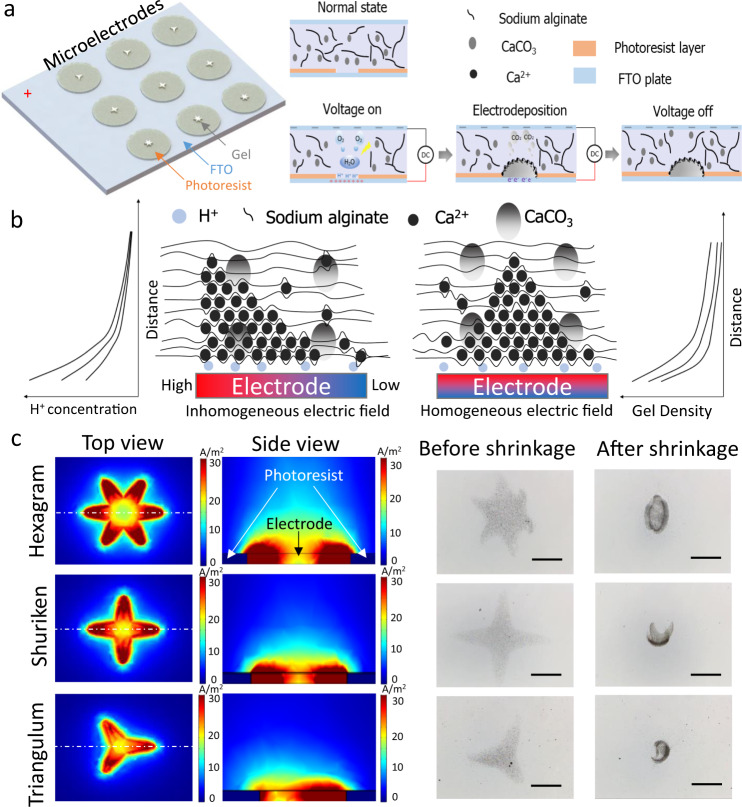
Principle of programmed shape deformation. **a** Fabrication of microelectrodes and ISMEs. The various patterned microelectrodes are fabricated by coating a layer of photoresist on the FTO glass and photolithography. And the ISME is fabricated through inhomogeneous electrodeposition. **b** Schematic of alginate hydrogel electrodeposition under different electric fields. Two figures represent that the H^+^ concentration and gel always decrease along the perpendicular direction. **c** Current density simulation and deformation of the corresponding alginate hydrogel hexagram, shuriken, and triangulum ISME microstructures. Through the design of the microelectrodes and the control of current density, the inhomogeneity electric field can be generated. Under inhomogeneous electrodeposition, the alginate microstructures are able to achieve shape morphing under pH or ionic stimulus. Scale bar: 500 μm.

We used a microelectrode-based method to fabricate the alginate hydrogel, as illustrated in Fig. 2a. Inspired by the morphing mechanism of the starfish, the pattern of hexagram, shuriken, and triangulum electrodes were fabricated through processing of photoresist on SnO_2_:F (FTO) plate as shown in Supplementary Fig. 1. The patterns of these electrodes mainly determine the electric field and facilitate localization of the electric field. In the normal state, the gap between the two plates was filled with the deposition solution. By applying a constant electric current on both sides of the FTO layers, oxygen and H^+^ ions were generated by water electrolysis. During the electrolysis reaction, the H^+^ ions were generated on the anode plate surface, which led to a rapid pH decrease and triggered the reaction forward to the electrodeposition process. In the electrodeposition process, the H^+^ ions reacted with CaCO_3_ particles to generate CO_2_ and Ca^2+^ ions which reacted with sodium alginate to achieve the Ca–alginate gelation. In the conventional electrodeposition, the homogeneous electric field results in an homogeneous gel network density as shown in Fig. 2b^31,32^. However, because of the edge effect on the microelectrodes, the microelectrode generated a heterogeneous electric field, resulting in an inhomogeneous gel network density. After the electrodeposition process, the alginate hydrogel microstructures remaining on the electrodes were detached and collected by washing with N-2-hydroxyethylpiperazine-N-2-ethane sulfonic acid (HEPES) buffer. These alginate microstructures were immersed in CaCl_2_ solution to shrink the sol–gel structure by crosslinking reaction with Ca^2+^, and then sodium citrate solution was pumped in to swell the gel structure through replacing the Ca^2+^. During this process, the higher difference of spatial gel crosslink network results in the higher deformation degree, which gives the alginate microstructure self-coiling and self-folding ability under ionically simulated swelling and shrinkage.

Figure 2c shows a simulation of a static low-frequency electric field with hexagram, shuriken, triangulum patterns. The tips of the patterns have higher current density, which causes a locally higher degree of cross-linking during electrodeposition. As a result, the difference in hydrogel density is the greatest between pairs of tips that are opposite one another. The inhomogeneous alginate hydrogel network will undergo shape deformation upon shrinkage and swelling in response to changes in pH or ionic strength. The factors like the CaCO_3_ concentration and deposition duration jointly determined the heterogeneity, which is challenging to be decoupled and quantify the corresponding effect to the crosslinking density under microscale. To demonstrate the relationship between electrode field and heterogeneity of gel spatial network more straightforward, the 3D simulation of H^+^ concentration (Supplementary Fig. 6) was built through fixing the value of the CaCO_3_ concentration, current density distribution, and deposition duration. The shape deformations of the hexagram (Supplementary Movie 1), shuriken (Supplementary Movie 2), and triangulum (Supplementary Movie 3) ISMEs also show that the tips of these radial alginate microstructures undergo a larger degree of deformation than the central part, which causes a folding transformation from the center to the tip.

### Self-transformation of ISMEs

**Fig. 3:**
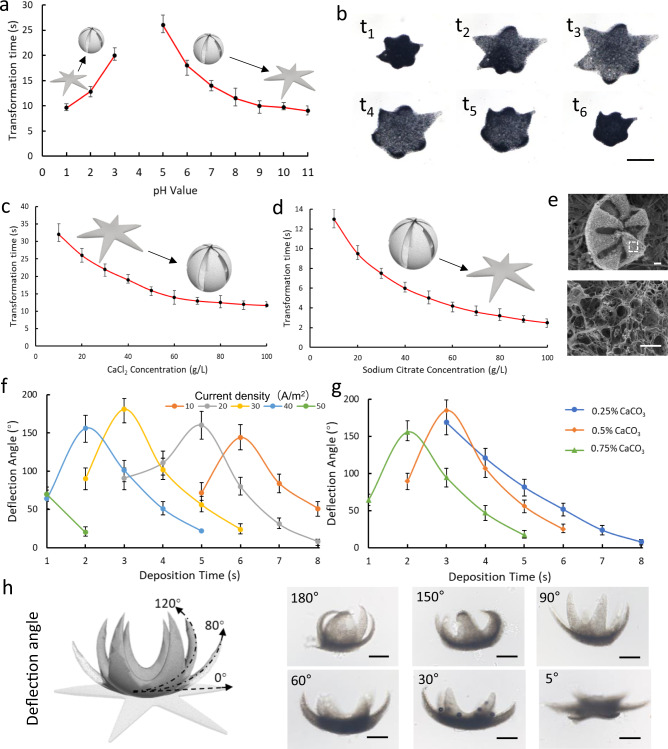
Process parameters of ISME transformation. **a** Time for an ISME to accomplish transformation in response to stimulation with different pH values. Each dot represents the average transformation time for five different ISMEs under pH shift with hexagram shapes ± s.e.m. (standard error of the mean). In the pH 1–4, the ISME represent the shrinkage. In the pH 5–11, the ISME represent the swelling. **b** Reversible transformation of ISME in response to pH changes. Scale bar: 500 μm. **c** Time for an ISME to accomplish shrinkage under different calcium ion concentrations. Each dot represents the average transformation time ISMEs with hexagram shapes ± s.e.m. *N* = 5. **d** Time for an ISME to accomplish swelling under different sodium citrate concentrations. Each dot represents the average transformation time ISMEs with hexagram shapes ± s.e.m. *N* = 5. **e** SEM images of the ISMEs. Scale bar: 10 μm. **f** Deflection angle of the finger part of an ISME formed with different current densities and deposition times. Each dot represents the average deflection angle structures with hexagram shapes ± s.e.m. *N* = 5. **g** Deflection angle of the finger part of an ISME formed with different calcium carbonate concentrations and different deposition times. Each dot represents the average deflection angle structures with hexagram shapes ± s.e.m. *N* = 5. **h** Images of an ISME with different deflection angles. The deflection angle represents the deflected angle from before to after deformation. Scale bar: 250 μm. Source data is provided as a Source Data file.

Controllable transformation is important for an ISME to perform tasks such as cell capture and target sampling. Thus, the speed of ISME transformation after pH change was evaluated, and the dependence of the transformation speed on the CaCl_2_ and sodium citrate concentrations. We observed that the ISMEs undergo shrinkage at pH 1–3 and swelling at pH 5–11, but no transformation occurs when the pH is around 4, as shown in Fig. 3a. Figure 3b and Supplementary Movie 4 illustrate the reversible shape transformation under a pH shift as HCl and NaOH are pumped in. All relevant data was provided in the Source Data. In this experiment, MNPs were encapsulated in the ISMEs to visualize the microstructures under a bright-field microscope. The ISME takes 32 s to complete the folding transformation at a CaCl_2_ concentration of 90 mmol/L (Fig. 3c). When the CaCl_2_ concentration was increased to 900 mmol/L, the transformation time decreased to 11 s. The same tendency was observed for the releasing motion: the ISMEs take 13 and 2.5 s to complete the release process at sodium citrate concentrations of 90 and 900 mmol/L, respectively (Fig. 3d). The cellular osmotic range in vivo is 290–310 mmol/L, which provides an ample range of ionic strengths to control the shape morphing and dissolution through ionic changes.

The scanning electron microscopy (SEM) images in Fig. 3e show that the ISME is well deformed into a gripper trap and that the pore size of the alginate hydrogel is ~1–10 μm after freezing. The ISME transformation triggered by different cross-linking densities in different regions of the alginate hydrogel mainly arose from differences in the CaCO_3_ concentration, current density, and electrodeposition time. The relationships among current density, deposition time, and deformation of the ISMEs are shown in Fig. 3f, g. Figure 3h shows the deformation degree defined as the measured deflection angle in the ISME, which was measured as the difference between the angles of the fingertip before and after deformation. The ISMEs exhibited the best deformation ability when the deposition time was 2–6 s, and the current density was 30 A/m^2^. The ISMEs were not able to fully gel when the deposition time was too short, and they exhibited impaired transformation ability if the deposition time was too long. As shown in Fig. 3f, g, the ISME shows no more shape morphing ability when the current density is above 50 A/m^2^ with 2 s electrodeposition duration (thickness over 290 μm before the shrinkage) or below 10 A/m^2^ with 5 s electrodeposition duration (thickness below 110 μm before the shrinkage). At a given current density (30 A/m^2^), the presence of CaCO_3_ at 0.5% (w/v) led to a better deformation ability than at 0.25% or 0.75% (w/v). A lower CaCO_3_ concentration produced less gelling over a short deposition time, whereas a higher concentration resulted in poorer deformation ability at a relatively longer deposition time.

### Mechanical properties of ISMEs

**Fig. 4:**
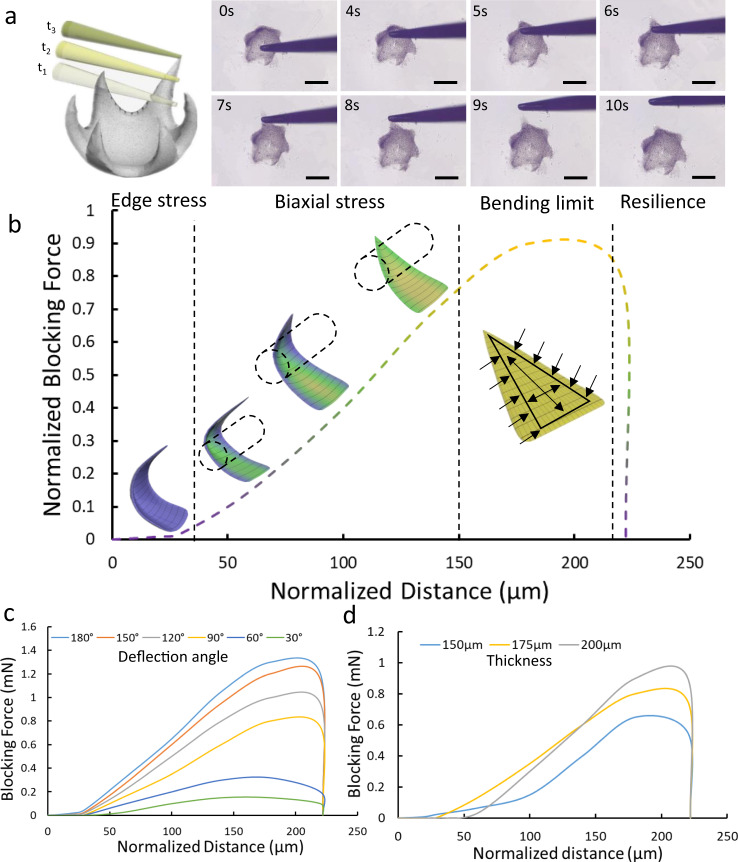
Mechanical test of an ISME. **a** Schematic illustration and images of the contact between the force transducer and the finger of an ISME at different distances. Scale bar: 250 μm. **b** Schematic illustration of ISME deformation and the ISME-generated strain force under elastic deformation. The tip of the force transducer firstly contacts with the edge of the ISME finger, which generate a small force named edge stress. And then, the tip of the force transducer contact with the long axis of the ISME finger. In this process, there are both edge stress and stress of long-axis, which is named biaxial stress. With the force transducer moving forward to the tip of the ISME finger, the force transducer give the bending limit and following the resilience of the ISME finger. The colouration of each state is arbitrary, which only gives the sequential process. **c** Blocking forces generated by ISME fingers with different deflection angles. **d** Blocking forces generated by ISME fingers with different thicknesses. Source data is provided as a Source Data file.

To determine the grip strength of an ISMEs the strain force of an ISME ‘finger’ was evaluated using a commercially available force transducer mounted on a 3D motorized precision translation stage. The probe of the force transducer was perpendicular to the finger and moved in the direction from ISME center to the tested tip parallel to the horizontal plane with the speed of 50 μm/s, as illustrated in Fig. 4a and Supplementary Movie 5. In this process, the finger was fully bent, and the blocking force was measured.

As shown in Fig. 4b, the modeling procedure included four steps to measure: (1) the edge stress at normalized distance *D* = 0 μm; (2) the biaxial stress at *D* = 30 μm; (3) the bending limit at *D* = 150 μm; and (4) the resilience at D = 220 μm. At *D* = 222 μm, the probe contacted both edges of the finger without the long axial deformation, which generated relatively low strain stress. With the probe moving forward at *D* = 30 μm, the finger underwent deformation not only on both edges, but also along the long axis. The long axial elastic force was much larger than the edge stress, because there was less deformation on the edge than along the long axis. At *D* = 150 μm, the probe was still moving toward the tip of the finger, and the force caused by the strain reached the bending limit of ~1 mN for each finger. In this process, the probe underwent the largest deformation, strain, and stress. After the probe moved beyond the tip at *D* = 220 μm, the finger sprung back, and the strain stress immediately decreased to zero. The deflection angle of an ISME indicates the extent of deformation of the finger, as shown in Fig. 4c; thus, ISMEs with larger deflection angles exhibit greater forces due to strain at the bending limit than those with smaller deflection angles, which have smaller edge stress. For ISMEs with deflection angles of 90°, thicker ISMEs have smaller edge stress than thinner ISMEs but generate higher forces along the long axis (Fig. 4d). Besides, the deflection angle of the ISME is related to the current density and deposition duration as illuminated in Fig. 3f. To explore the relationship between the current density and structural mechanical properties, we selected deflection angle 120°, current density 30 A/m^2^ and CaCO_3_ concentration 0.5% to simulate the finger structural stress of the ISME as a qualitative result reflecting the influence of the local mechanical properties to the deformation of the structure (Supplementary Fig. 7) . Through comparing the simulation result with the current density simulation (Fig. 2c), the spatial current density distribution is positively related to the structural stress which contributes to the ISME shape deformation.

### MNPs encapsulated ISMEs and active transportation

**Fig. 5:**
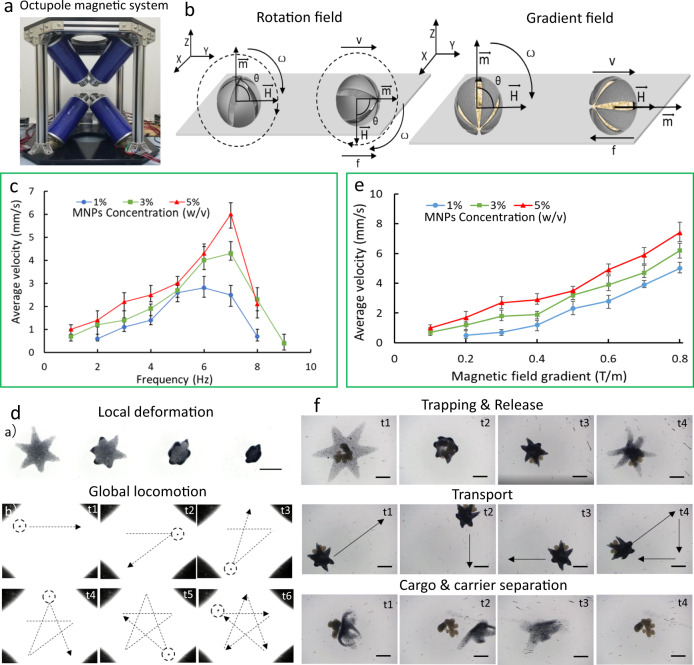
Manipulation of an ISME with encapsulated MNPs under different magnetic fields. **a** Octupole magnetic system used in the experiment. **b** Schematic illustration of the magnetic actuation of an ISME under a rotating field and a gradient field, wherein *V* represents the translational velocity, **H** denotes the strength of the magnetic field, *m* indicates the direction of magnetization of the ISME, *f* represents the force of friction, *ω* indicates the rotation velocity of the microsphere, and *θ* denotes the tilt angle between the direction of the magnetic field and the direction of magnetization of the ISME. **c** Velocity–frequency profiles for ISMEs with different MNP concentrations (1%, 3%, and 5%) under a rotating magnetic field. Each dot represents the average velocity for five different ISMEs with hexagram shapes ±s.e.m. (standard error of the mean). **d** (a) Images of the ISME with encapsulated MNPs during folding transformation in response to a pH change. Scale bar: 500 μm. (b) Time series of the rotating-field-controlled locomotion of an ISME with time instants marked and motion trajectories delineated. Scale bar: 4 mm. **e** Velocity–frequency profiles of ISMEs with different MNP concentrations (1%, 3%, and 5%) under a gradient magnetic field. Each dot represents the average velocity for five different ISMEs with hexagram shapes ±s.e.m. (standard error of the mean). **f** Images of MNP-loaded ISMEs performing the capture, transportation, and release of cell aggregates, separation following release, and eventually dissolution in response to a pH change. Scale bar: 250 μm. Source data is provided as a Source Data file.

To achieve target capture and sampling, MNPs were encapsulated in the ISMEs to provide transportation under a magnetic field. The transportation and navigation of MNPs loaded ISMEs were achieved using an octupole electromagnetic system, as shown in Fig. 5a. This electromagnetic system can generate two types of magnetic fields: a rotation field and a gradient field. Figure 5b represent ISME actuation under both fields. Controlled locomotion is influenced by the MNP concentration and current frequency, as shown in Fig. 5c. At frequency 1–7 Hz, the ISME rotation is synchronized with the applied rotation field, and the ISME velocity increases almost linearly with the field frequency. After reaching a maximum velocity at 7 Hz, the velocity decreases as the field frequency increases further because the available magnetic torque is no longer sufficient to keep the ISME synchronized with the applied field. In addition, the velocity was positively correlated with the MNP concentration. Figure 5d and Supplementary Movie 6 demonstrate efficient ISME deformation and locomotion under pH values from 7 to 1 in a magnetic field with 5 Hz rotation when the MNP concentration was 3% (w/v). Due to the irregularity of the ISME, there is an off-course (maximum ±3 mm) in the magnetic manipulation, which can be reduced by a motion control algorithm and a flexibly changeable magnetic field.

The rotation field is applied to achieve the global transportation, which has superiority on less input current to actuate microrobot, and the gradient field is conveniently applied to maintain the posture in local environment. Figure 5e shows that the ISME velocity is generally proportional to the MNP concentration and the magnetic field gradient. In one application, a gradient magnetic field can be used to control ISMEs with encapsulated MNPs to trap cells, transport the cells to a target area, release the cells in response to a pH change, and separate from the cells by magnetic manipulation, as illustrated in Fig. 5f and Supplementary Movie 7. In the envisioned applications, the clinically precious cells should be maintained by supplying with necessary biological and mechanical cues^33^. The ISME is also able to provide a mechanical maintenance through constantly pushing the captured cells towards the target location during the cell delivery.

### Magnetic manipulation and passive transportation of ISMEs

**Fig. 6:**
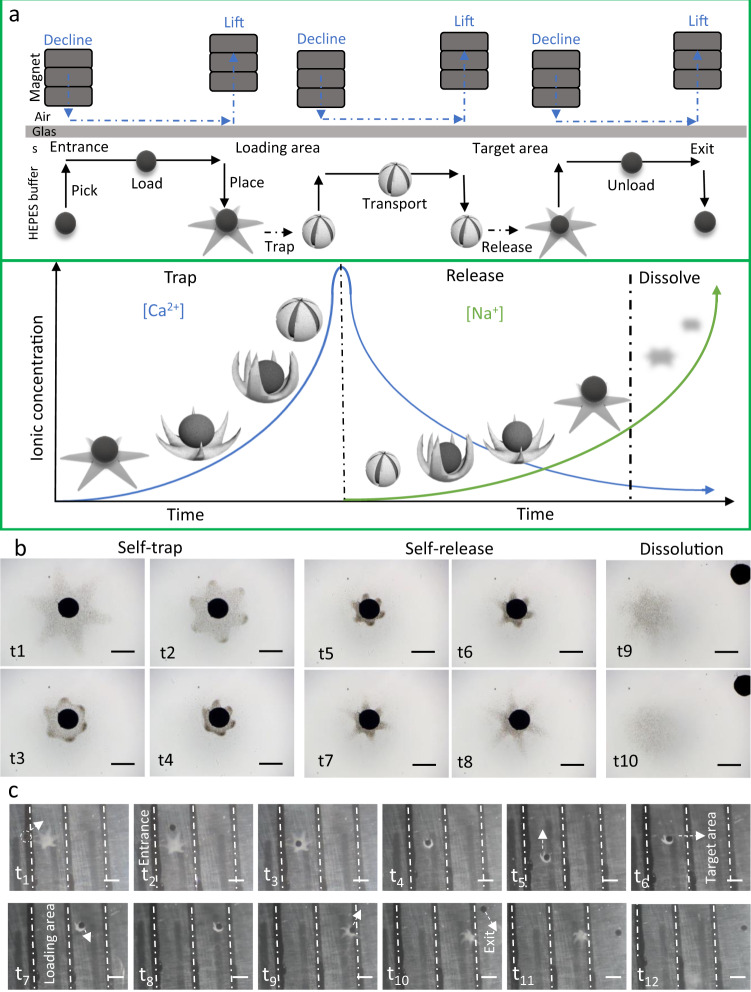
Magnetic manipulation of an ISME through self-trapping magnetic microspheres. **a** Illustration of the cargo transportation task and corresponding ISME transformations under ionic stimulation. **b** Images of ISME self-trapping a magnetic microsphere upon application of a CaCl_2_ solution and self-releasing the magnetic microsphere upon application of a sodium citrate solution followed by full dissolution. Scale bar: 500 μm. **c** Side-view images of the cargo transportation task in HEPES buffer. Scale bar: 1 mm.

A biomedical microrobot is expected to arrive at the target area with high spatial precision and conduct a series of tasks without undesirable physiological effects. Passive transportation provides safer drug or cell delivery than active transportation. To achieve passive transportation, a magnetic manipulation system was used to place PEGDA magnetic microspheres onto the ISME, as illustrated in Fig. 6a and Supplementary Movie 8. Then, a 1% (w/v) CaCl_2_ solution was pumped in, which caused the magnetic microspheres to self-trap in the ISME. Thus, the ISME and magnetic microsphere formed a tight assembly to accomplish transportation from a leading area to a target area under control by a magnetic field. Subsequently, a sodium citrate solution (1% (w/v)) can be applied to stimulate self-release and separation from the magnetic microspheres, which can then be removed through the exit of the magnetic manipulation system. Although the ISME was fully dissolved, the encapsulated cargo could be delivered and distributed to the target area which has large potential in the in vivo applications. Figure 6b shows the folding, releasing, and dissolution process during delivery. All procedures and trajectories of the ISME delivery are illustrated in Fig. 6c.

### In vitro self-release and cell delivery

**Fig. 7:**
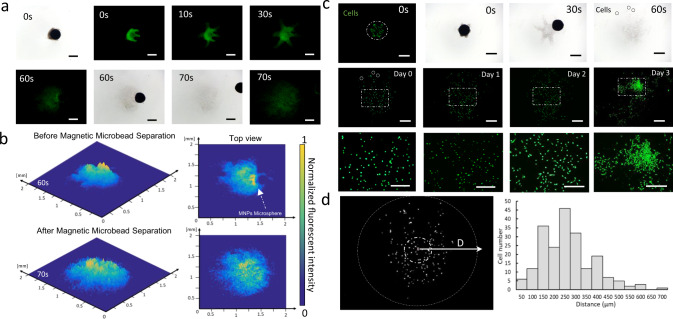
In vitro delivery test. **a** Bright-field and fluorescent images showing the distribution of fluorescent nanobeads encapsulated in an ISME and magnetic microsphere removal at 70 s. Scale bar: 500 μm. **b** Intensity maps representing the distribution of fluorescent nanobeads before and after magnetic microsphere separation at 60 and 70 s, respectively. **c** Bright-field and fluorescent images illustrating the dissolution of fluorescent-stained cells encapsulated in the ISME and magnetic microsphere removal at 30 s; cell proliferation is shown over 3 days of cultivation. Scale bar: 500 μm. **d** Gray-scale image representing the cell distribution on day 0 and quantification of the distances of the cells from the distribution center, which is evaluated in the histogram. Scale bar: 500 μm. Source data is provided as a Source Data file.

To investigate the releasing process of ISMEs, fluorescent nanobeads (diameter: 500 nm) were used as markers to better visualize the ISME transformation. An artificial colonic fluid was used to mimic the native environment of the intestine and to dissolve the ISMEs in HEPES buffer. Figure 7a and Supplementary Movie 9 show the release of the ISME from a magnetic microsphere driven by magnetic manipulation, and the distribution of fluorescent nanobeads distribute before and after magnetic microsphere separation. The distribution of fluorescent nanobeads was analyzed in MATLAB. From the results shown in Fig. 7b, the magnetic microsphere prevents the spreading of fluorescent nanobeads at 60 s. After the removal of the magnetic microsphere under magnetic manipulation, the fluorescent nanobeads were evenly distributed in a 1-mm diameter circle at 70 s. In addition, fluorescent-stained cells were encapsulated in the ISME to evaluate the cell distribution. As shown in Fig. 7c and Supplementary Movie 10, after the releasing of the ISME, the magnetic microsphere was removed from distributed area to ensure the cells were evenly distributed. Based on these results, over 80% of the cells were distributed in the range of 150–300 μm from the ISME center, and the cell viability was above 85% (Supplementary Fig. 5). After 3 days of culture, the cell viability remained high, and cell proliferation was observed. The cell distribution results were analyzed in OpenCV, as shown in Fig. 7d. First, a distribution center was determined as the point that has the shortest distance to every single cell. This calculated distribution center was compared with the ISME center to ensure the cell distribution results were not influenced by liquid flow or other environmental factors.

### Ex vivo manipulation of the ISME

**Fig. 8:**
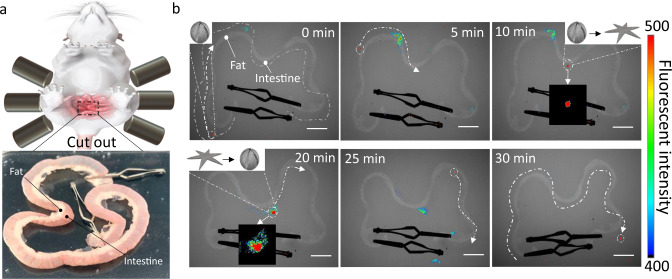
Magnetic actuation of the ISME and ISME transformation in the intestine ex vivo. **a** Ex vivo model of a rat intestine. The rat intestine was cut out from the SD rat before the ex vivo test. **b** Ex vivo transformation and transportation of ISME carrying MNPs and fluorescent microbeads controlled by an external magnetic field. Scale bar: 2 cm.

The ex vivo manipulation of the ISME was demonstrated in a rat intestine as shown in Fig. 8a. The intestine was extracted from a Sprague Dawley rat and immersed into an artificial stomach fluid. An ISME with an encapsulated fluorescent bead was injected into the intestine, and both ends of the intestine were sealed using an arterial clip. In vivo imaging (Fig. 8b) shows that the ISME was initially located on the left end of the intestine. Under the magnetic field, the ISME was moved along the intestine to the middle part, where the artificial intestinal liquid was injected near the ISME location. In response to this stimulus, the ISME transformed and self-released within 10 min, during which the fluorescence signal was higher than that in the shrunken state of the ISME. After 20 min, a CaCl_2_ solution was injected near the ISME to enable it to self-trap and transform back to its former structure. The ISME was then moved farther to the right of the intestine. These results confirm the ability of the ISME to conduct long-distance locomotion and transformation in a physiological environment. In the potential in vivo application, the common operations like the apastia and ionic solution intake can be performed and a 90 mmol/L CaCl_2_ solution is totally enough and safe for the patient to maintain the stability of the ISME.

## Discussion

We developed environmentally adaptive ISMEs with the motions of gripper for targeting, releasing, and sampling in vitro and ex vivo. The ISMEs were fabricated by electrodeposition of alginate hydrogel in the size range of 600–200 μm (after the shrinkage) and facilitated motion of grasp which is stimulated by pH or ionic changes. Generally, the heterogeneous electric field endows the alginate hydrogel architectures with different crosslinking densities during the electrodeposition. Non-crosslinked carboxylate groups in the electrodeposited alginate hydrogel can be further reacted with various multivalent cations (e.g. Ca^2+^, Ba^2+^, Cu^2+^ and Al^3+^, etc.)^31,32,34^, in which the shrinkage/swelling of alginate is governed by the ion-exchange process taking place between Na^+^ ions and multivalent cation (Ca^2+^ especially) in the ISMEs^33,35,36^. During the hydrogel shrinkage process stimulated by pH or ionic changes, the local differences in cross-linking density led to in-plane stress, resulting in inhomogeneous shape deformation. This in-plane stress can be released through swelling (i.e., expansion) of the hydrogel network when the pH or ionic change is reversed. When the alginate hydrogel kept in its swelled state, the hydrogel network will over-expand and eventually disintegrate and completely dissolve. Before the over-expansion of the hydrogel network, the hydrogel can still undergo the grasp-release transformation under the proper ionic or pH environment^37^. The shape of the microelectrode can be designed to create various patterns of hydrogel cross-linking density in the alginate hydrogel microstructures, giving rise to different shape-morphing abilities in the fabricated ISME.

The ISMEs can be propelled by active or passive transportation for grasping and delivery tasks, respectively. Active transportation of the ISMEs with encapsulated MNPs was achieved using rotating and gradient magnetic fields, which can be selected based on the specific requirements of the application environment. Because of the small size of the ISMEs, the rotating motion was more efficient than that generated by pulling using the gradient magnetic field and, thus, was more suitable for long-distance locomotion^38–41^. However, the gradient field provided gentler locomotion, which is more suitable for maintaining the desired posture when grasping the target and transportation of living samples without impacting their viability. In passive transportation, bioactive materials (e.g., cells or drugs) can be embedded in the ISMEs instead of MNPs, and magnetic microspheres can be self-trapped as “hitch-hikers”^9^. Through the manipulation of magnetic microspheres under a magnetic field, the ISMEs can be precisely navigated to the target area and deliver their bioactive cargo through self-release and dissolution. In the current stage, the viscosity of water (1 mPa s) is 100 times smaller than stomach liquid (100 mPa s) which leads to the 10 times smaller viscous drag^42^. However, the current magnetic field strength (3–20  mT) is able to be promoted to 0.35–3 T (the magnetic field strength of clinical MRI system), which represents the 1000 times larger magnetic field force. For the free locomotion of ISMEs, the force condition in potential application (magnetic driving force vs. viscous drag) is still over 10 times larger comparing with current magnetic field strength and liquid environment. Besides, the MNPs concentration and liquid environment of GI tract can be improved through apastia and drinking. Since the pH value of GI experiences a change from pH = 1 in stomach to pH = 7 in intestinal tract, the ISMEs conduct shrinkage and grasping in the stomach followed by release and even dissolution as they proceed to the intestine. Further, there are ionic solutions (Ca^2+^, sodium citrate, etc.) are able to adjust and control these swelling and shrinkage process in practical application. After the delivery process, the magnetic microspheres can be removed under the magnetic field and excreted with no release of cytotoxic material, including the MNPs. As one application of these capabilities, we demonstrated that ISMEs can be used as movable microgrippers with active transportation to achieve precise sampling ex vivo under various ionic environments in the GI tract.

The in vitro and ex vivo tests of the ISME is performed to further verify the feasibility of the fabrication method and robotic design, which demonstrate the possible potential in future applications. The in vitro experiments demonstrated that the ISMEs with encapsulated MNPs can grasp, release, and transport cells. The ex vivo experiments demonstrated locomotion and shape-morphing under the various physiological environments. Thus, the ISMEs have potential to conduct more complex tasks in the human digestive system if the samples and ISMEs can be visualized in real time by real-time via imaging^43^. However, the development of such an in vivo imaging modality is still in the early stages, so it remains a challenge to obtain precise real-time 3D visualization and localization for precise manipulation of the ISMEs in in vivo fluidic environments. To improve the efficiency, the control of MNPs loaded multi-ISMEs is under exploration. This will allow simultaneously multiple target capture (as shown in Supplementary Fig. 2 and Supplementary Movie 11), which has potential in biomedical and physiological research.

In conclusion, we demonstrated the use of an alginate hydrogel monolayer as an environmentally adaptive ISME that can conduct cell delivery and target sampling under stimulation by changes in the pH or ionic conditions. Delivery and sampling processes were demonstrated in vitro and in an ex vivo organ, which mimics the in vivo environment. This work provides a logical, rapid technique for targeted cell delivery and target capture using ISMEs. This approach has implications for future applications in minimally invasive medicine, precise diagnosis, and manufacturing of smart materials and smart surfaces.

## Methods

### Materials

FTO-coated glass was purchased from Huanan Xiangcheng Inc. (Shenzhen, China). Dulbecco’s modified Eagle’s medium with phenol red, fetal bovine serum (FBS), trypsin, penicillin, streptomycin, and phosphate-buffered saline were obtained from Gibco (Carlsbad, CA, USA). NIH/3T3 fibroblasts were purchased from ATCC (Manassas, VA, USA). Calcein-acetoxymethyl ester and propidium iodide were purchased from Dojindo Molecular Technologies Inc. (Kumamoto, Japan). MNPs (Ni nano particles) were purchased from Beijing Zhongkeleiming Technology Co. Ltd. (Beijing, China).

### Production of microelectrode

To conduct electrodeposition, microelectrodes were designed as shown in Supplementary Fig. 1. The microrobots were designed using a computer-aided design tool (SolidWorks, Dassault Systèmes, SolidWorks Corp. Inc., USA). Different shapes were designed for fabricating different microstructures. The electrode fabrication method was mainly based on photolithography. Firstly, a 10-μm layer of photoresist (AZ5214) was coated on the surface of 50 mm × 50 mm FTO glass using a spin coater. The photoresist-coated FTO glass was then baked on a hotplate for 1 min at 100 °C. Then, a homogeneous ultraviolet light was passed through a predesigned photomask onto the photoresist-coated FTO glass. This FTO glass was then developed in an AZ developer for 45 s, and 10-μm-thick patterns were achieved. The patterned FTO glass was then baked on a hotplate for 2 min at 120 °C to fix the photoresist for use as electrodes.

### Heterogeneous electrodeposition of alginate hydrogel

To produce an alginate monolayer, a deposition solution of 1% (w/v) sodium alginate and 0.25%, 0.5%, or 0.75% (w/v) CaCO_3_ particles was used. The 1%, 3%, or 5% (w/v) MNPs, 2% (v/v) fluorescent nanobeads and cells were mixed in the deposition solution before the electrodeposition. The maximum MNPs load capacity is below 7% (w/v). Firstly, 2 mL deposition solution was dropped onto the anode plate between two electrodes. Secondly, a 3–5 V direct current voltage was applied on both FTO layers of the electrodes for 1–10 s. After the electrodeposition process, the anode plate was washed with HEPES buffer in a 10 cm Petri dish for over 3 min until the calcium alginate hydrogel microstructures were completely detached from the electrode. When the Ca–alginate microstructures had been fully removed, these alginate microstructures were transferred to another 6 cm Petri dish containing HEPES buffer. CaCl_2_ solution (1.1% w/v) and sodium citrate solution (1% w/v) were slowly injected until the microstructures had fully transformed. The images were taken by Olympus IX83 microscope under bright field. And the size of the ISME was measured through Olympus IX83 microscope under bright field. Before the magnetic manipulation, the ISME was magnetized along the horizontal line direction of the ISME with the external magnetic strength (13 mT) to form remanence in the directional arrangement of the nickel nanoparticles, so as to maximize the magnetic moment and driving force. The magnetization cure of the MNPs encapsulated ISME is provided in Supplementary Fig. 4.

### Mechanical test of microrobot

To measure the elastic force of each finger on the ISME, mechanical tests were conducted using a Model #405A force transducer system purchased from Aurora Scientific Inc. This force transducer system was assembled on a 3D motorized precision translation stage driven by stepping motors (model NSA12; New Focus Inc.) to achieve 3D motion during the measuring process. A glass probe was fixed on the end of force transducer, and the probe has a 50 μm tip on the back-end to contact with the ISME. The force transducer system was moved along the direction from the ISME center to the tested tip parallel to the horizontal plane with the speed of 50 μm/s. The thickness of ISMEs was evaluated by scanning the fluorescent microbeads encapsulated ISME under confocal microscope (A1, Nikon, Japan) before the shrinkage. The ISME deflection angle was evaluated before the strain force experiment by analyzing bringht-field images of the ISME captured using an Olympus IX83 microscope.

### Electromagnetic system

Taking both the required magnetic rotating field and gradient field ISME into account, an octupole electromagnetic system was developed. The 100-mm workspace was surrounded by eight electromagnetic coils with DT4 magnetic cores, distributed in the diagonal directions. The imaging system consisted of a miniature camera (MD028MU-SY, XIMEA) with a frame size of 1934 × 1456 pixels and a lens (FA2502D, CHIOPT) with a focal length of 25 mm. The computer–microcontroller communication determined the programmable digital current of each coil, through the corresponding amplifier (ESCON 50/5, MAXON, Sachseln, Switzerland), with the PID feedback, ranging in ±5 A with a resolution of 0.3 mA. To generate the desired magnetic field, all currents can be calculated in real-time by the inverse matrix, according to the linear superposition of their contribution to the magnetic field. For rotating field, the magnetic components in three directions was solved by the rotation matrix. When the current amplitude is 1 A, it can realize a rotating field with the magnitude of 20 mT and the maximum frequency of 20 Hz. For gradient field, opposite currents were input into the adjacent coils to realize the gradient of 0.3 T/m when the current amplitude was 1 A.

### Passive transportation

The magnetic manipulation system was composed of a three degree-of-freedom magnetic manipulation system with a gradient magnetic field (the magnetic strength is shown in Supplementary Fig. 8), the magnetic microspheres and alginate ISME (in HEPES buffer in a 6-cm Petri dish), and a side-view microscope (HDC1400C, Panasonic, with 2–4 times variable telephoto lens) to monitor the delivery process, as shown in Fig. 5c. In the Petri dish (diameter: 6 cm with a cover glass sheet diameter: 5 cm), three black lines divided the area into four regions: the entrance, loading area, target area, and exit. The distance between each line was 3 mm. An array of disk magnets (*B*_max_ = 0.2 T, diameter: 3 mm) was vertically installed under the slider of the manipulator. Depending on the pulley and lead screw drive, the slider enabled the magnet to move along the *x* and *z* directions with a positioning resolution of 0.01 and 0.004 mm, respectively. In addition, the mobile platform moved along the *y* direction with a resolution of 0.01 mm. To prevent ISME drift by liquid flow, the solution pump-in speed was 500 μL/min.

The manufacturing process of magnetic microspheres is illustrated in Supplementary Fig. 3. First, we mixed 2% (w/v) photoinitiator (IRGACURE 2959) and 5% (w/v) MNPs into PEGDA solution (molecular weight: 700 g/mol), all of which were purchased from Dojindo Molecular Technologies Inc. (Kumamoto, Japan). Then, PEGDA solution was dropped in the mineral oil using a pipette with a 50-μm-diameter tip. The PEGDA droplet was exposed to UV light (wavelength: 365 nm) for 20 s.

### Cell counting

The number of cells embedded in each microrobot was counted using ImageJ software. After the ISME was dissolved, the cells were evenly distributed at the bottom of the Petri dish. The distance between the ISME center and each cell was measured using OpenCV. The data suggest that over 80% of cells were distributed in the range of 150–300 μm from the center of the ISME.

### Ex vivo tests

We used Sprague–Dawley rats (male, about 250 g in weight) that were purchased from the MaiDiSiWei Inc., Beijing, China. All surgical and experimental procedures were approved by the Institutional Animal Care and Use Committee of the Beijing Institute of Technology, Beijing, China. Mice were housed on a 12/12 h light/dark cycle at a consistent ambient temperature (23 ± 1 °C) and humidity (50 ± 5%), and all experiments were performed during the light cycle. Food and water were accessed ad libitum.

Rats were euthanized using excessive anesthesia (intraperitoneal injection of 2% sodium pentobarbital at a dose of 40 mg/kg). The abdominal cavity was then opened, and the digestive organs from the esophagus to the end of the rectum were excised. A 15 cm length of the intestines was removed beginning 1 cm from the stomach and immersed in artificial stomach liquid (Solarbio, Beijing, China). The fluorescent microbeads and MNPs loaded ISME (red, diameter: 1 μm, PolySciences Inc., USA) was injected into the intestine, and the ends of the intestine were then sealed using two arterial clips. The ISME was manipulated using the magnetic system. After the ISME was moved to the middle part of the intestine, 1 mL of artificial intestinal liquid was injected near the ISME’s position. After observation for 5 min, 1 mL of 1% (w/v) CaCl_2_ solution was injected near the ISME. The manipulation process was observed using a combined in vivo-imaging system designed for small animals (In-Vivo FXPro, Bruker) with an X-ray scan time of 60 s and optical excitation and emission of 510 and 600 nm, respectively.

### Reporting summary

Further information on research design is available in the Nature Research Reporting Summary linked to this article.

## Supplementary information

Supplementary Information

Description of Additional Supplementary Files

Supplementary Movie 1

Supplementary Movie 2

Supplementary Movie 3

Supplementary Movie 4

Supplementary Movie 5

Supplementary Movie 6

Supplementary Movie 7

Supplementary Movie 8

Supplementary Movie 9

Supplementary Movie 10

Supplementary Movie 11

Reporting Summary

## Data Availability

All data related to this manuscript is available from the corresponding authors upon reasonable request.  Source data are provided with this paper.

## Source data

Source Data
