# Author Correction: Ionic shape-morphing microrobotic end-effectors for environmentally adaptive targeting, releasing, and sampling

**DOI:** 10.1038/s41467-021-21986-8

**Published:** 2021-03-05

**Authors:** Zhiqiang Zheng, Huaping Wang, Lixin Dong, Qing Shi, Jianing Li, Tao Sun, Qiang Huang, Toshio Fukuda

**Affiliations:** 1 Intelligent Robotics Institute, School of Mechatronical Engineering, Beijing Institute of Technology, 100081 Beijing, China; 2 Beijing Advanced Innovation Center for Intelligent Robots and Systems, Beijing Institute of Technology, 100081 Beijing, China; 3 Department of Biomedical Engineering, City University of Hong Kong, 999077 Hong Kong, China; 4 Key Laboratory of Biomimetic Robots and Systems (Beijing Institute of Technology), Ministry of Education, 100081 Beijing, China

**Keywords:** Biomedical engineering, Mechanical engineering, Bioinspired materials

Correction to: *Nature Communications* 10.1038/s41467-020-20697-w, published online 18 January 2021.

The original version of this Article contained an error in the author affiliations.

Lixin Dong was incorrectly associated with ‘Beijing Advanced Innovation Center for Intelligent Robots and Systems, Beijing Institute of Technology, 100081 Beijing, China.’ instead of the correct ‘Department of Biomedical Engineering, City University of Hong Kong, 999077 Hong Kong, China’.

In addition, the following statement was omitted from the ‘Acknowledgement‘ section:

‘L.D. thanks the Beijing Institute of Technology for hosting him as a visiting professor prior to join the City University of Hong Kong, which has enabled this collaborative work’.

These errors have now been corrected in both the PDF and HTML versions of the article.

