# Supplementary material for: Ionic shape-morphing microrobotic end-effectors for environmentally adaptive targeting, releasing, and sampling
Source: Nat Commun. 2021 Jan 18;12:411. doi: 10.1038/s41467-020-20697-w (PMC7814140; doi:10.1038/s41467-020-20697-w)
Supplement: Supplementary file 1 — Supplementary Information [file 41467_2020_20697_MOESM1_ESM.pdf]

Supplementary Information

Ionic Shape-morphing Microrobotic End-effectors for Environmentally Adaptive Targeting, Releasing, and Sampling

Zhiqiang Zheng[1], Huaping Wang*[,2], Lixin Dong[2], Qing Shi[3], Jianing Li[1], Tao Sun[2], Qiang Huang*[,2,3], Toshio Fukuda[3]

Affiliations

1. Intelligent Robotics Institute, School of Mechatronical Engineering, Beijing Institute of Technology, Beijing 100081, China.
2. Beijing Advanced Innovation Center for Intelligent Robots and Systems, Beijing Institute of Technology, Beijing 100081, China.
3. Key Laboratory of Biomimetic Robots and Systems (Beijing Institute of Technology), Ministry of Education, Beijing 100081, China.

* Corresponding author. Email: wanghuaping@bit.edu.cn (H.W.); qhuang@bit.edu.cn (Q.H.)

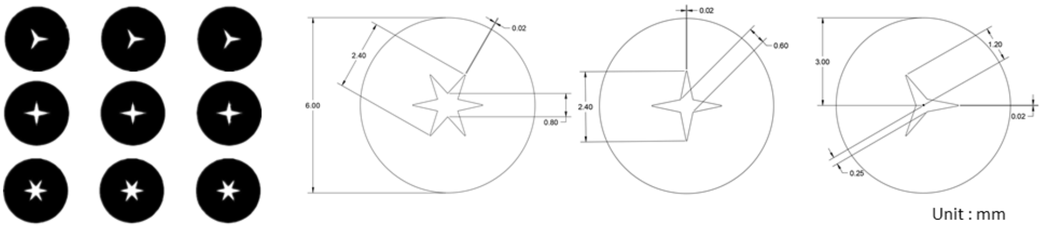

Supplementary Fig. 1. The design and parameter of various electrodes. The black regions are the areas of remained photoresist, and the white regions are the areas of exposed FTO layer which are used as electrodes.

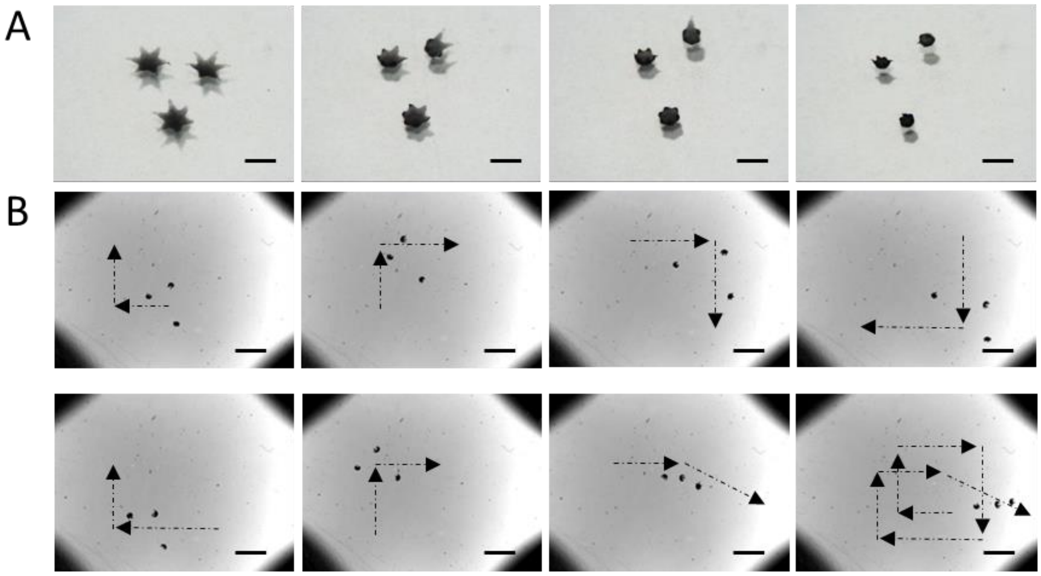

Supplementary Fig. 2. The process of multi-ISMEs self-trapping and locomotion. (A) The self-trapping motion of three ISMEs stimulated by $CaCl_2$. Scale bar: 500 µm (B) The trajectory of multi-ISMEs under rotational magnetic manipulation. Scale bar: 3 mm

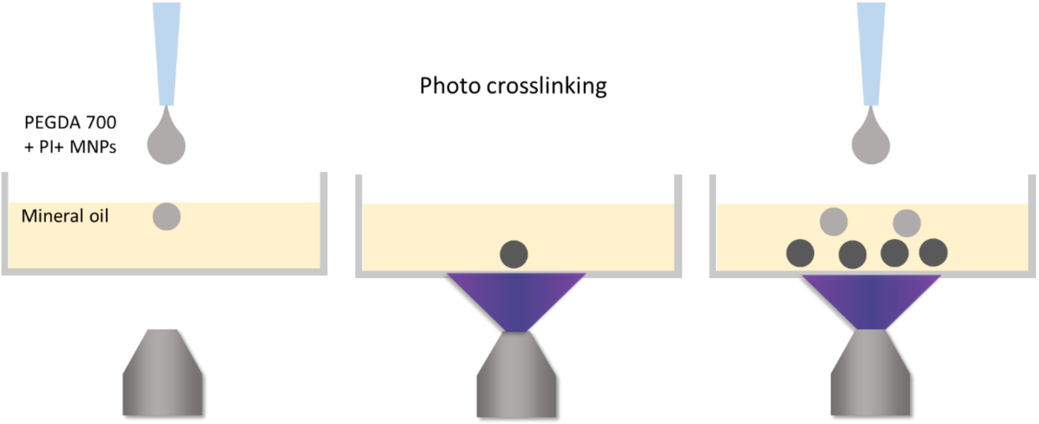

Supplementary Fig. 3. The process of magnetic microspheres manufacture.

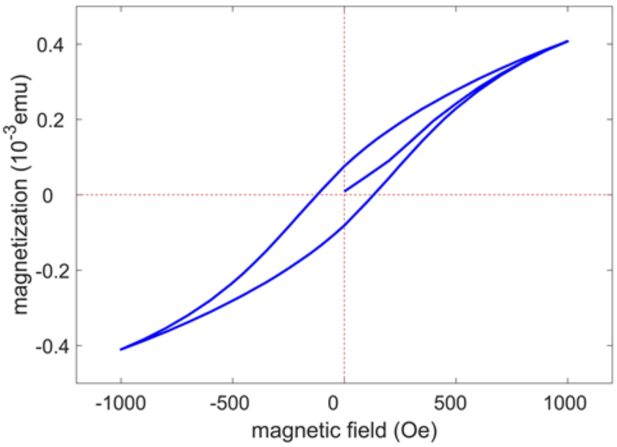

Supplementary Fig. 4. Magnetization of the MNPs encapsulated ISME.

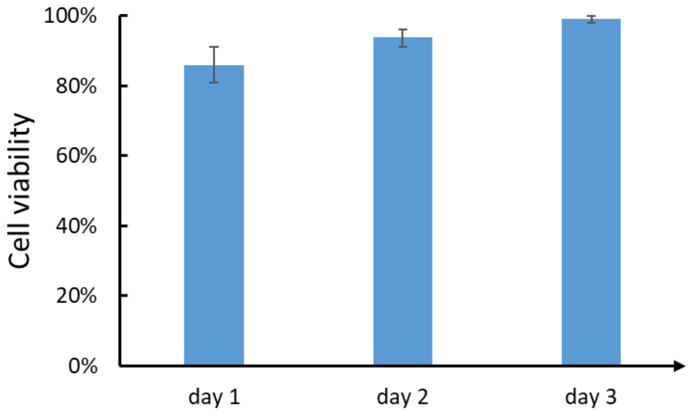

Supplementary Fig. 5. Cell viability test after the passive propulsion of the ISME delivery. Each bar represents the cell viability for five different ISMEs delivery results with hexagram shapes ± s.e.m. (standard error of the mean).

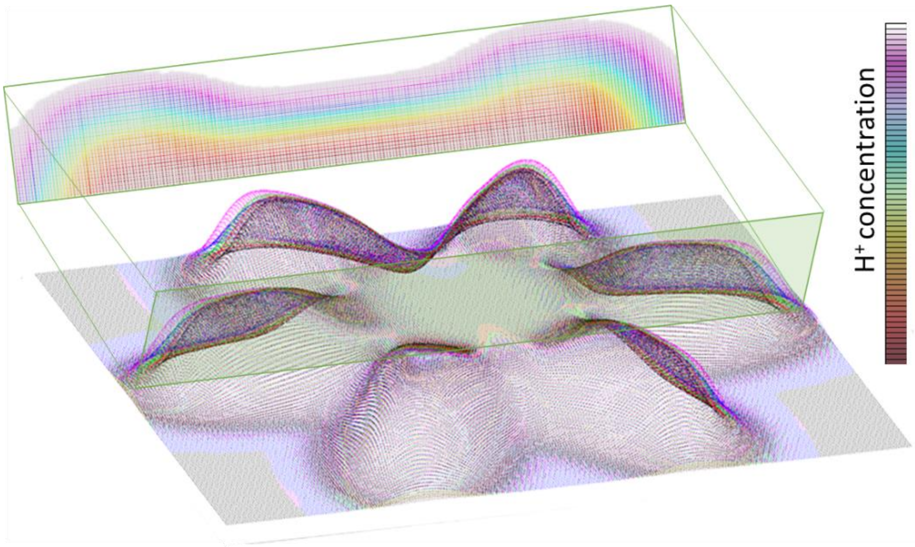

Supplementary Fig. 6. 3D simulation of H^+ concentration.

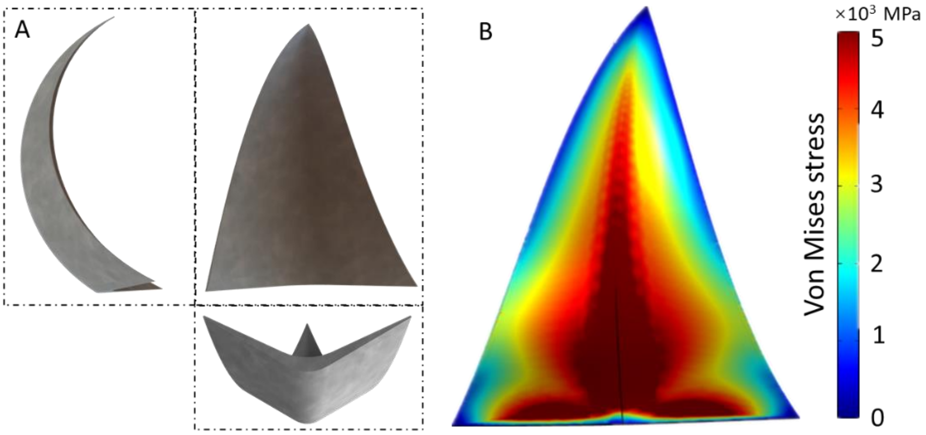

Supplementary Fig. 7 (A) 3D finger model setup. (B) Finger structural stress simulation of the ISME.

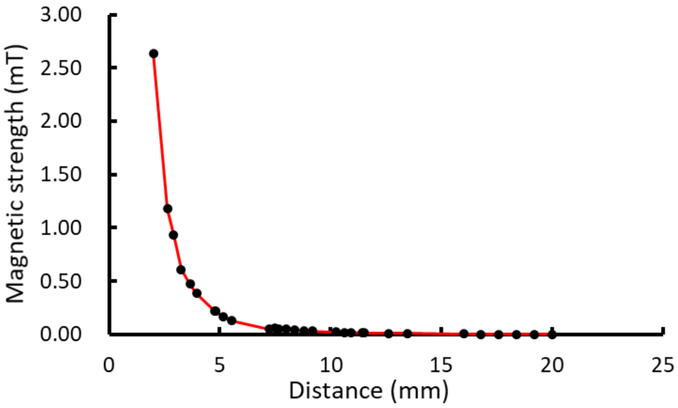

Supplementary Fig. 8. The magnetic strength various of the distance to the surface of magnet in the passive transportation.
