# Supplementary material for: Ionic shape-morphing microrobotic end-effectors for environmentally adaptive targeting, releasing, and sampling
Source: Nat Commun. 2021 Jan 18;12:411. doi: 10.1038/s41467-020-20697-w (PMC7814140; doi:10.1038/s41467-020-20697-w)
Supplement: Supplementary file 2 — Description of Additional Supplementary Files [file 41467_2020_20697_MOESM2_ESM.pdf]

Description of Additional Supplementary Files

Supplementary Movie 1.

Shape transformation of hexagram microrobot.

Supplementary Movie 2.

Shape transformation of shuriken microrobot.

Supplementary Movie 3.

Shape transformation of triangulum microrobot.

Supplementary Movie 4.

Reversible shape transformation of microrobot in response to pH shift.

Supplementary Movie 5.

Grip strength test of microrobot.

Supplementary Movie 6.

Active propulsion of magnetic nanoparticles encapsulated microrobot.

Supplementary Movie 7.

Active propulsion of microrobot with encapsulated MNPs performing the capture of cell aggregates, transportation, release, separation and eventually self-dissolution.

Supplementary Movie 8.

Passive propulsion of microrobot was achieved by self-trapping magnetic microspheres as hitch-hikers.

Supplementary Movie 9.

Self-release process of microrobot visualize by fluorescent nanobeads.

Supplementary Movie 10.

Fluorescent stained cells were encapsulated in the microrobot to evaluate the cell distribution in in vitro test.

Supplementary Movie 11.

Control of MNPs encapsulated multi-microrobots.
