# Supplementary material for: Ionic shape-morphing microrobotic end-effectors for environmentally adaptive targeting, releasing, and sampling
Source: Nat Commun. 2021 Jan 18;12:411. doi: 10.1038/s41467-020-20697-w (PMC7814140; doi:10.1038/s41467-020-20697-w)
Supplement: Supplementary file 14 — Reporting Summary [file 41467_2020_20697_MOESM14_ESM.pdf]

nature research

Corresponding author(s): Huaping Wang,Qiang Huang

Last updated by author(s): 2020/11/19

Reporting Summary

Nature Research wishes to improve the reproducibility of the work that we publish. This form provides structure for consistency and transparency in reporting. For further information on Nature Research policies, see our Editorial Policies and the Editorial Policy Checklist.

Statistics

For all statistical analyses, confirm that the following items are present in the figure legend, table legend, main text, or Methods section.

n/a	Confirmed	
☐	☒	The exact sample size (n) for each experimental group/condition, given as a discrete number and unit of measurement
☐	☒	A statement on whether measurements were taken from distinct samples or whether the same sample was measured repeatedly
☐	☒	The statistical test(s) used AND whether they are one- or two-sided<br>*Only common tests should be described solely by name; describe more complex techniques in the Methods section.*
☐	☒	A description of all covariates tested
☐	☒	A description of any assumptions or corrections, such as tests of normality and adjustment for multiple comparisons
☐	☒	A full description of the statistical parameters including central tendency (e.g. means) or other basic estimates (e.g. regression coefficient) AND variation (e.g. standard deviation) or associated estimates of uncertainty (e.g. confidence intervals)
☐	☒	For null hypothesis testing, the test statistic (e.g. F, t, r) with confidence intervals, effect sizes, degrees of freedom and P value noted<br>*Give P values as exact values whenever suitable.*
☒	☐	For Bayesian analysis, information on the choice of priors and Markov chain Monte Carlo settings
☒	☐	For hierarchical and complex designs, identification of the appropriate level for tests and full reporting of outcomes
☐	☒	Estimates of effect sizes (e.g. Cohen's d, Pearson's r), indicating how they were calculated

Our web collection on statistics for biologists contains articles on many of the points above.

Software and code

Policy information about availability of computer code

Data collection	SolidWorks 2016 Comsol Multiphysics 2017 Auto CAD 2016 MATLAB R2016b Premiere Pro CC2018 OriginLab OriginPro 8.5 GraphPad Prism 8.0
Data analysis	static electric field simulation Von Mises stress simulation: Comsol Multiphysics Confocal imaging data Statistical Analysis: GraphPad Prism 8.0 3D model design:SolidWorks 2016 2D shape design:Auto CAD 2016 Imaging analysis:MATLAB R2016b

For manuscripts utilizing custom algorithms or software that are central to the research but not yet described in published literature, software must be made available to editors and reviewers. We strongly encourage code deposition in a community repository (e.g. GitHub). See the Nature Research guidelines for submitting code & software for further information.

Data

Policy information about availability of data

All manuscripts must include a data availability statement. This statement should provide the following information, where applicable:

- Accession codes, unique identifiers, or web links for publicly available datasets
- A list of figures that have associated raw data
- A description of any restrictions on data availability

All the relevant data used to prepare the manuscript and the Supplementary Information.
All data will be made available on request to the corresponding author.

Field-specific reporting

Please select the one below that is the best fit for your research. If you are not sure, read the appropriate sections before making your selection.

- [x] Life sciences
- [ ] Behavioural & social sciences
- [ ] Ecological, evolutionary & environmental sciences

For a reference copy of the document with all sections, see nature.com/documents/nr-reporting-summary-flat.pdf

Life sciences study design

All studies must disclose on these points even when the disclosure is negative.

Sample size	The size of the ISME was measured through Olympus IX83 microscope under bright field. The length of the rat intestine was determined by a straight steel ruler.
Data exclusions	There is no data exclude from analysis.
Replication	We did the same experiments five times. Based on the experiment, the organ sample always suitable for the magnetic manipulation as mentioned in the manuscript.
Randomization	We randomly allocate the mice.
Blinding	The intestine of the mice just provide a ex vivo environment which is no need for blinding.

Reporting for specific materials, systems and methods

We require information from authors about some types of materials, experimental systems and methods used in many studies. Here, indicate whether each material, system or method listed is relevant to your study. If you are not sure if a list item applies to your research, read the appropriate section before selecting a response.

Materials & experimental systems

n/a	Involved in the study
x	Antibodies
	x Eukaryotic cell lines
x	Palaeontology and archaeology
	x Animals and other organisms
x	Human research participants
x	Clinical data
x	Dual use research of concern

Methods

n/a	Involved in the study
x	ChIP-seq
x	Flow cytometry
x	MRI-based neuroimaging

Eukaryotic cell lines

Policy information about cell lines

Cell line source(s)	NIH/3T3 fibroblasts
Authentication	The NIH/3T3 fibroblasts cell line was purchased from ATCC (Manassas, VA, USA).
Mycoplasma contamination	Tested and found negative for ectromelia virus (mousepox), which is provided by ATCC. Cell line was not tested for mycoplasma contamination by our own.
Commonly misidentified lines (See ICLAC register)	This is the only one cell line used in this study.

Animals and other organisms

Policy information about studies involving animals; ARRIVE guidelines recommended for reporting animal research

Laboratory animals	We used Sprague–Dawley rats (male, about 250 g in weight) that were purchased from the MaiDiSiWei Inc., Beijing, China. All surgical and experimental procedures were approved by the Institutional Animal Care and Use Committee of the Beijing Institute of Technology, Beijing, China. Mice were housed on a 12/12 h light/ dark cycle at a consistent ambient temperature (23 ± 1 °C) and humidity (50 ± 5%), and all experiments were performed during the light cycle. Food and water were accessed ad libitum.
Wild animals	Did not involve wild animals

Field-collected samples	The study did not contain field-collected samples.
Ethics oversight	All experimental procedures were approved by the Institutional Animal Care and Use Committee of Beijing Institute of Technology

Note that full information on the approval of the study protocol must also be provided in the manuscript.
